# The Search for the Elusive Basic Processes Underlying Human Intelligence: Historical and Contemporary Perspectives

**DOI:** 10.3390/jintelligence10020028

**Published:** 2022-05-13

**Authors:** Robert J. Sternberg

**Affiliations:** Department of Psychology, College of Human Ecology, Cornell University, Ithaca, NY 14853, USA; rjs487@cornell.edu

**Keywords:** components of intelligence, general intelligence, individual differences, intelligence, metacomponents, basic processes, theories of intelligence

## Abstract

This article discusses the issues of the basic processes underlying intelligence, considering both historical and contemporary perspectives. The attempt to elucidate basic processes has had, at best, mixed success. There are some problems with pinpointing the underlying basic processes of intelligence, both in theory and as tested, such as what constitutes a basic process, what constitutes intelligence, and whether the processes, basic or not, are the same across time and space (cultural contexts). Nevertheless, the search for basic processes has elucidated phenomena of intelligence that the field would have been hard-pressed to elucidate in any other way. Intelligence cannot be fully understood through any one conceptual or methodological approach. A comprehensive understanding of intelligence requires the converging operations of a variety of approaches to it.

## 1. Introduction

While a paradigm in science is active and “hot”, it seems as though it will provide definitive answers that some previous paradigm failed to provide (Kuhn 2012). Time passes, and as Kuhn pointed out, the new paradigm illustrates the notion that the solution to one problem merely becomes the next problem to be solved.

The search for the basic processes of intelligence—which once seemed like it might somehow “solve” the problems of the test-based psychometric approach—has proven to be a case in point for Kuhnian thinking. It showed how, in the history of psychology, an approach that at one time seems like it may provide some kind of ultimate solution, becomes merely the next set of problems to be solved. For those who believe that any current approach to intelligence—such as a neuropsychological or cultural approach—will somehow provide a kind of final resolution, the history of the process-based approach might contain a warning. Contrary to what some might hope, there is no plausible end to paradigms, any more than there is a hoped for “end” to history (Fukuyama 2006). Yet, each paradigm elucidates phenomena that previous paradigms failed adequately to elucidate.

The search for the basic processes underlying intelligence began not in psychology but rather in philosophy (see Sternberg 2020a). Plato suggested in Book 5 of the *Republic* that the basic process of intelligence is learning: a more intelligent person learns faster. More intelligent people also show a love of learning absent in the less intelligent. Aristotle suggested, in the *Posterior Analytics*, that the processes of syllogistic reasoning are the keys to intelligence. Furthermore, philosophers continued to argue, after the ancient philosophers, about what intelligence is. Perhaps the most well-known philosopher of intelligence was Kant, who suggested in the *Critique of Pure Reason* that intelligence comprises three processes: understanding, judgment, and reason.

Why would anyone even want to understand the basic processes, as opposed to anything else, underlying intelligence? Consider three reasons: First, basic processes reveal a temporal course of cognition in a way that other units, such as factors, typically do not (Sternberg 1985b)—how people solve problems from beginning to end. Second, basic processes sometimes reveal a confounding in prior beliefs about intelligence (Carroll 1976; Hunt 1980). For example, a test that might have been believed to measure “verbal reasoning” might, through process analysis, be revealed mostly to be a vocabulary test (Sternberg 1987). Furthermore, even those who have leaned heavily on psychometric theorizing often have sought to supplement such an analysis with an information-processing analysis (e.g., Jensen 1998; Spearman 1923). Third, an information-processing analysis provides a useful complementary perspective on other kinds of analyses, such as neuropsychological and psychometric analysis. Arguably, there is no one ideal or somehow uniquely correct level of analysis for understanding intelligence—different levels can tell investigators different things about intelligence, which is why handbooks on the topic review research encompassing a broad range of approaches (e.g., Goldstein et al. 2019; Sternberg 2020c).

The information-processing approach in psychology—the attempt to identify basic processes—occurred in reaction to earlier trends in psychology. Behaviorism, at least in its radical form, rejected any analysis of internal mental processing, focusing instead on the analysis of observable behavior (Skinner 1965). Gestaltism was concerned with inner processing but at a very global rather than specific and basic level (e.g., Köhler 1970). For example, calling something “insight” did not provide a lot of insight into what insight is (Duncker 1972). Furthermore, psychoanalysis was much more concerned with unconscious and motivated processing than with conscious information processing (Freud 1989).

The search for the basic processes of intelligence was motivated and inspired, at least in part, by the fundamental early works of information-processing psychology. The first was Miller et al. (1960), which proposed the TOTE (test–operate–test–exit) as the fundamental unit of information processing. The second was Neisser (1967), which proposed analysis-by-synthesis as the basis of information processing. Furthermore, the third was Newell and Simon (1972), which proposed the eip (elementary information process) as the fundamental unit. Hunt et al. (1973) saw this approach as an opening to a new view on human intelligence.

There is no one broadly accepted notion of what a “basic” process even is. In a so-called MOVE problem, as theorized about by Newell and Simon (1972) and by Jeffries et al. (1977), among others, it is one move that takes a problem solver from one step of a set of discrete steps in a well-defined problem space to the next step. In Sternberg’s (1983) componential theory of intelligence, building on Spearman’s (1923) analysis, it is a discrete, serial, mental step in solving an intellectual problem for which one can ascertain a reaction time and error rate. Sternberg (1983), similar to Jensen (1982a, 1982b), Hunt (1980), and others, calculated process time through the subtraction method, subtracting reaction time for processing one stimulus from reaction time for processing another that was alleged to differ only by one additional discrete identifiable process. Fry and Hale (1996) emphasized the importance of processing speed, whatever the process, an emphasis shared by Jensen and others. In Jensen’s case, the emphasis was on the speed of neuronal conduction, and Haier (2016) has also pointed to such speed as important to understanding intelligence. 

The differences in views, which continue to the present day, call into question whether the construct of a *basic process* even has any clearly specifiable psychological meaning other than the meaning a researcher stipulates for it. On the one hand, “basic” seems to be in the mind of the beholder. On the other hand, some stipulated meanings have had more long-term heuristic value than others. For example, Spearman’s (1923) processing ideas have greatly affected much future research; that said, ideas about MOVE problems were in great currency for the latter part of the twentieth century but are less influential today because they seem more limited in their application to well-defined problems with clear and unique or nearly unique steps and paths to solution. Thus, although there is no one universally “correct” definition of *basic processes*, some meanings have generated more research over a longer period of time than have others.

## 2. Major Contentions of this Article

This article makes three major contentions:

1. The search for basic processes of intelligence has had, at best, mixed success because researchers do not know how to find truly basic processes. They would not even know if they found the basic processes because there is no empirical test that will reveal a set of processes as basic. Nor is it even clear what a “basic process” is. As a result, much of the literature is searching for a kind of “grail” that, if it existed, would not be recognized if it were found.
2. The attempt of researchers of intelligence to argue for the superiority of their information-processing analysis of intelligence based on levels of correlation coefficients is misguided and fruitless for five reasons.
  - As we all learn in elementary statistics, correlation does not imply causation.
  - Correlations of process measures with measures of intelligence tend to be modest or at best moderate, in any case (see essays Sternberg 1984, 2020c).
  - Even when the correlations are moderate, the direction of causality is unclear: for example, the correlations may be due to hidden third variables.
  - Although the information-processing approach was originally designed to redefine intelligence research and move beyond psychometrically-based correlational studies (e.g., see, Hunt et al. 1973; Sternberg 1977), the correlations of information-processing measures with psychometric tests assume, somewhat ironically, that conventional psychometric tests are an appropriate ultimate criterion for whether the information-processing analysis is valid. As a result, the information-processing analyses become subservient to the psychometric analyses they originally were designed to improve upon and perhaps even ultimately to replace.
  - Meta-analyses of the strength of relation between information-processing measures and psychometrically measured intelligence (see, e.g., Grudnik and Kranzler 2001; Jensen 1998; Kranzler and Jensen 1989; Redick and Lindsey 2013) do not address the question of basic processes because, for the most part, they combine results of correlational analyses, and thus are themselves correlational analyses. That is, combining correlational analyses still leaves one with a correlational analysis.
3. There are now so many measures that have been found to correlate significantly with measured intelligence, more or less in line with Spearman’s (1904, 1927) findings, that any claims of causality are largely useless because correlations already have shown themselves not to discriminate well among alternative claims regarding what is basic (see Conway and Kovacs 2020; Ellingsen et al. 2020; Nettelbeck et al. 2020). If statistically significant correlations signify a winner of the “basic process” derby, then there are too many winners of this derby.

In sum, it is not clear that any claims about “basic information processes” are falsifiable. Significant correlations of process latencies or error rates with intelligence-test scores do not necessarily imply any particular process is somehow “basic”, and even where correlations are not significant, one cannot draw firm conclusions from null correlations. Moreover, given that some theories go beyond what is measured by traditional psychometrically motivated intelligence tests (e.g., Gardner 2011; Sternberg 2021a, 2021b), it may be that there are processes that are somehow “basic,” just not to the processing that psychometric tests measure. 

Now, let us review where these contentions come from.

## 3. Historical Perspectives on the Processes Underlying Intelligence

Historically significant psychological work on basic processes underlying individual differences in intelligence started with psychometricians.

### 3.1. Psychometric Origins

In psychology, the search for the basic processes underlying intelligence goes back at least to Galton (1883/1907/1973), who sought to understand intelligence through psychophysical processes, such as detecting faint sounds or differences between pitches. The attempt, carried on by James McKeen Cattell’s lab at Columbia University, was not successful. Psychophysically-based measures of supposedly basic processes correlated neither with each other nor with grades at Columba University (Wissler 1901). Studies with better instruments for measurement, more trials per participant, and modern data analysis have shown such measures to produce some statistically significant correlations, usually modest to moderate (see reviews in, e.g., Deary 2000; Haier 2016; Jensen 1998). However, it is still not clear what the correlations mean, as stated earlier.

A different attempt at elucidating processes, but not necessarily “basic” processes of intelligence, can be traced to Binet and Simon (1916). They proposed that intelligence involves judgement, good sense, practical sense, initiative, and fitting oneself into one’s environment (pp. 42–43). Binet and Simon’s approach often has been viewed as atheoretical (e.g., White and Hall 1980), because the test based on the approach was constructed on the basis of selection of items that discriminated empirically across chronological ages. However, this criticism is not really fair. Binet and Simon (1916) suggested that three processes underlie intelligence (see Sternberg 1990, 2020b):

- **Direction.** This process involves knowing what has to be accomplished and how it can be accomplished. Today it might be called something like “problem formulation.”
- **Adaptation.** This process requires selecting, implementing, and monitoring one’s strategies for problem solving so as to maximize adaptation to the environment.
- **Control.** This process involves the ability to critique and correct one’s thoughts and actions. It is a reflective process that ensures one can regulate how one solves a problem.

Binet’s successor as the creator of a major intelligence test, David Wechsler, took a position very similar to Binet’s, although his intelligence test was somewhat different (Wechsler 1944). Wechsler, too, believed that intelligence resides largely in the ability to adapt to the environment and to negotiate one’s position in the everyday world (Wechsler 1940). 

On the one hand, Binet and Simon did propose a theory. On the other hand, it would be difficult to say that it was a theory of basic processes. For example, adaptation may be a process, but it almost certainly is not a “basic process.” What constitutes adaptive thinking or behavior depends upon the situation in which one finds oneself, so the processes that constitute adaptation will also be determined situationally, at least in part (Sternberg 2021a). Similarly, control may be a process, but it is hard to see how it could be “basic” or, in Newell and Simon’s (1972) terminology, “elementary”. One would need to create a thought, and then then figure out what is wrong with it, and then critique what is wrong with it, and then correct the thought. It is worth noting that Newell and Simon used the term “elementary” to describe the processes they elicited, but they no more showed that the processes are elementary than modern intelligence researchers have shown their processes are elementary or basic. These problems apply as well to the early work of the author of this article (Sternberg 1977, 1981, 1983, 1985a).

Charles Spearman in England was less interested in commercially useful intelligence tests and more interested in the theory of intelligence. In his work on the general factor of intelligence, Spearman (1923) identified three processes of intelligence that he believed were the keys to individual differences. These processes can be identified in analogical reasoning, such as in the analogy *dark : light :: tall : ?*

- **Apprehension of experience.** This process is what we today might call encoding, as in reading the terms of the analogy and understanding what they mean;
- **Eduction of relations.** This is an inferential process, as in recognizing the relation between *dark* and *light;*
- **Eduction of correlates.** This process involves applying the relation one has inferred, as in applying the relationship of antonyms to recognize the opposite of *tall* is *short.*

Spearman’s work on the general (*g*) factor of intelligence (Spearman 1927) gained much more attention than did his work on the mental processes underlying intelligence. However, neither line of work went very far in elucidating elementary information processes underlying intelligence. Obviously, *g* was intended to be only a composite construct. Spearman (1927) believed it to be a result of “mental energy”, but it never was very clear what mental energy was, and it certainly did not seem to be a basic process but rather some utilized reserve of motivation. Furthermore, the three processes may have been useful in solving analogies or other inductive-reasoning problems, but they were far from a comprehensive model of basic intellectual processes. Indeed, calling the “eduction of relations” the same for simple analogies and for complex world problems, such as the causes of war, would seem to be a stretch too far.

After Spearman, the quest for an understanding of the processes underlying intelligence went somewhat into remission as psychometrically oriented researchers emphasized structural rather than process-based models. There were a few exceptions, however.

Thorndike (1911) sought to understand animal intelligence in terms of associationist principles, which also could be applied to humans, as humans are animals. Thurstone (1924), in one of his lesser-known works, related intelligence to overcoming instinctual responses and replacing them with more reflective responses. Interestingly, Stenhouse (1973) came to the same conclusion as Thurstone, using evolutionary rather than information-processing or psychometric analysis. Stenhouse suggested that what differentiates species and individuals within species intellectually is their ability to inhibit instinctive responses. Those organisms that consistently act on the basis of what Kahneman (2011) later would call Type 1, or impulsive thinking, are less likely to survive because they fail to see the long-term consequences of actions that are not, in some way, thought through. However, these models are all models of types of thinking, not of basic processes.

Guilford (1967; Guilford and Hoepfner 1971) made mental operations, that is, processes, one of the three facets of his structure-of-intellect (SI) model. The six mental operations posited by Guilford were

- **Cognition**—the ability to understand or comprehend;
- **Memory recording**—the ability to encode information and enter it into memory;
- **Memory retention**—the ability to remember information;
- **Divergent production**—the ability to generate multiple solutions to a problem lacking any one so-called “correct” answer;
- **Convergent production**—the ability to come upon a correct answer to a problem;
- **Evaluation**—the ability to judge whether an answer is correct or, at least, reasonable.

Guilford’s attempt was scientifically questionable (Horn 1967; Horn and Knapp 1973) because it turned out that, using Guilford’s method of “Procrustean rotation” of factors, (a) random data could produce good fit to Guilford’s psychological theory and (b) random theories could produce equally good fit to Guilford’s data. The Horn (1967) and Horn and Knapp (1973) analyses did not actually show Guilford’s theory to be “wrong”. Rather, they showed that what had appeared to be empirical support for the theory was much weaker than it previously had appeared to be. Nevertheless, Guilford’s work proved to be an important transition between psychometric and information-processing approaches to understanding intelligence. The problem was that, because the factor analyses on which the model was based were invalid, the model faded as an overly ambitious attempt by a scholar to bite off much more than he could chew. The eventual idea of 180 statistically independent abilities (Guilford 1982) just did not seem plausible, much less empirically supported. 

### 3.2. Early Information-Processing Psychology

With the publication of Miller et al. (1960) and Newell and Simon (1972), it was only a question of time before the new paradigm made its way into intelligence research. The call for the integration of psychometric research and its parent discipline, differential psychology, with experimental psychology came only seven years after the publication of Miller et al. (1960). Lee Cronbach (1957), in his presidential address to the American Psychological Association, called for an integration of differential and experimental approaches to psychology. His way of doing so was to be through so-called aptitude–treatment interaction (ATI) studies, through which one tried to match instructional treatments to pattens of aptitudes in students. Cronbach published a major work with Richard Snow (Cronbach and Snow 1977) on this approach. Unfortunately, the book was full of null results. Attempts to study aptitudes experimentally through ATI studies just did not seem to be working. The attempt to identify basic processes through ATIs did not succeed.

The first scholar successfully to employ the new paradigm may have been Royer (1971), who conducted an information-processing analysis of the digit-symbol substitution task on the Wechsler adult intelligence scales (Wechsler 1944). However, Royer’s approach was to do individual studies rather than to generate a more general theoretical and methodological approach to the information-processing analysis of intelligence. Furthermore, the choice of task may have been unfortunate because the task was one that, in the Wechsler tests, is factorially remote from general intelligence (Gignac and Vernon 2003). Royer did not identify, nor claim to identify, basic information processes.

#### 3.2.1. The Cognitive-Correlates Approach

A more general information-processing framework for studying intelligence was proposed by Hunt et al. (1973) and then followed up by Hunt et al. (1975). The approach came to be called the “cognitive-correlates” approach. The basic logic was that the underlying processes of intelligence could be identified by linking scores on cognitive tasks to those on psychometric tests, a proposal also made by Carroll (1976). Experimental participants would receive a cognitive task, such as the Posner and Mitchell (1967) letter-identification task. In this task, individuals would see a pair of letters and have to say either whether they are the same physically (e.g., T, T) or the same only in name (e.g., T, t). The difference in reaction time between name and physical match was taken as the time needed for the individual to retrieve the name of the letter from long-term memory. Hunt and colleagues also investigated other tasks, such as the S. Sternberg (1966, 1969) short-term memory-scanning task. Hunt’s work, however, was not the only cognitive-correlates-based work. 

Jensen (1982a, 1982b) proposed that intelligence might be understood in terms of the speed of neuronal conduction. He used an apparatus to measure choice reaction time that he devised to study the relation of mental speed to intelligence and found that choice reaction time was a predictor, if a somewhat modest one, on scores of psychometric tests of intelligence. Nettelbeck and Lally (1976), Deary and Stough (1996), and Vickers and Smith (1986) suggested the use of inspection time, which was a measure of accuracy. Participants would see two lines and be asked which line was longer. The measure of inspection time depended on how short a duration of exposure of the lines the participants consistently needed to determine which line was longer.

Hunt and his colleagues, and others as well, found that shorter reaction times were associated with higher scores on tests of verbal abilities. The correlations ran at about the −0.3 level, with the correlations negative because faster processing was associated with higher ability. Correlations were higher when corrected for attenuation and restriction of range. The scientists suggested that letter-identification time might be a basis.

The work of the cognitive-correlates researchers was seminal but, as is the case for most breakthrough seminal work, also problematical.

First, the cognitive-correlates researchers had the same problem the psychometricians had. They started with mental-ability tasks and then constructed theories around those tasks. The theories could be no better than the tasks. Hunt and colleagues followed the same procedure. They started with experimental tasks and then tried to build up post hoc theories around the tasks. The problem was that the theories were limited by the tasks that, in this case, were originally chosen by investigators for reasons other than to understand intelligence. The theories thus were ad hoc results of research, rather than the research being strongly theory-driven. In later work, Hunt (1980) related intelligence to the ability to engage effectively in dual-processing tasks (usually, a primary task and a distracting task), but again, the research appears primarily to have started from the experimental tasks rather than from a theory of intelligence. In each case, a plausible theory could be offered post hoc, but it derived largely from the experimental tasks that were used. 

Second, it never was quite clear what the theory was. For example, speed of lexical retrieval may be related to verbal ability, as may be speed on many other verbal tasks. However, a weak to moderate single empirical correlation does not a theory make. Other parameters of information-processing tasks also showed correlations with scores on tests of intelligence, but their theoretical significance was also not clear. Essentially, we are being asked to observe a correlation between a cognitive task and a psychometric test and conclude that one or more of the mental processes behind the task leads causally to individual differences in performance on the tests. That is a bit of a stretch. The same problem would apply to the work linking choice reaction time to general intelligence (e.g., Jensen 1998) or to the relationship of inspection time to general intelligence (Deary and Stough 1996; Nettelbeck and Lally 1976; Nettelbeck et al. 2020). The theories essentially followed from isolated correlations, however often replicated.

Third, it never was clear what the correlations between parameters of cognitive tasks and scores on cognitive tests meant. Although we all learn in elementary statistics that correlation does not imply causation, causal inferences seem to have been made to psychometric tests that have been alleged, but never shown conclusively, to measure intelligence. It is not clear that either the psychometric test or the cognitive task was in any sense causal. Both may have depended on some unidentified third element that underlies general intelligence. 

Fourth, it never was clear what level of correlation would count as being practically, as opposed to statistically, meaningful. Most of the correlations in the voluminous literature on cognitive correlates of intelligence-test scores are roughly at the 0.3 level. These correlations can be goosed up by various corrections, such as for attenuation and restriction of range, but those corrections are hypothetical, no matter how good they may make researchers and readers feel. For example, what is the “correct” range? Is it with an IQ 100 and a standard deviation of 15? However, that is an average that represents no particular population anyone is likely to deal with in real-world pursuits. College students have a higher average IQ with a reduced standard deviation. Candidates for executive positions likewise have higher average IQs and lower standard deviations. Prison populations probably have lower average IQs, as do populations of people who are not schooled in schools that teach abstract-reasoning skills. What one has is the 0.3 correlation, accounting for a bit less than 10% of the variance in the criterion. Is that enough to infer causality? Would a 0.5 correlation, accounting for 25% of the variation in the criterion, corrected for attenuation and restriction of range, be enough? 

It has become clear in the literature that almost any cognitive task correlates, without correction, 0.1 to 0.4 with IQ-related tests (Hunt 2011; Mackintosh 2011; Sternberg 2020c). With enough participants, the correlation is statistically significant. It is possible, of course, that if one combines all the scores for multiple components from various research studies, one will raise the multiple correlation coefficient to a point that looks “causal”. Detterman (1980) suggested, essentially following in the path of Thomson (1916), that there are many independent elements that together form general intelligence (*g*). Conway and Kovacs (2018) proposed a related theory, process-overlap theory (POT).

Fifth, some of the theoretical claims about the correlations were dubious. For example, Jensen (1998) claimed that choice reaction time is an indirect measure of neuronal conduction speed, but is it? Was the way people retrieved letter names in the Posner and Mitchell (1967) task identical or even similar to the way they retrieved letter names when the letters were embedded in meaningful words in real-world verbal contexts (Reicher 1969; Wheeler 1970)? Sometimes, abstracting a process from its real-world context may change the process or how it is executed.

Sixth, it never was quite clear what constituted a “process”, much less, a “basic process”. Did subtracting one reaction time from another yield a true estimate of a single process? Or might it have yielded a time for multiple processes that occurred in conjunction with each other? How could researchers ever know that what they isolated was a single process? These approaches have been helpful in elucidating processes, but the processes do not seem to resemble the elementary information processes sought by Newell and Simon (1972).

Seventh and finally, this approach assumes that intelligence tests should be the criteria and ultimate arbiters of whether a particular task measures “intelligence”. However, the tasks used to measure intelligence on intelligence tests were not selected because they satisfied some theoretical constraint on what such tasks should be. Rather, they were selected by Binet and Simon (1916) because, loosely, they seemed to measure reasoning and judgment skills and showed age differences among test-takers in success toward reaching the answers Binet and Simon deemed as “correct”. 

There are many *g*-based tasks that correlate with each other. However, whether collectively they comprise all that there is to intelligence is not at all clear. Indeed, the Flynn (1987, 2012, 2016) effect appears to be due in large part to a non-*g*-related variance, suggesting that even IQ involves much more than just *g* (Wicherts et al. 2004; Woodley et al. 2014. )Furthermore, intelligence involves more than IQ (Wechsler 1940).

#### 3.2.2. The Cognitive-Components Approach

Sternberg (1977) proposed a different approach to identifying the processes underlying intelligence, based upon his 1975 dissertation. Sternberg called the approach “componential analysis”, and it also sometimes was called “cognitive-components analysis”. Whereas Hunt selected tasks that were used in laboratory-based, information-processing studies of perception and cognition, Sternberg selected tasks that were used on psychometric tests to study intelligence. He started with analogies and then moved on to a variety of inductive and deductive reasoning tasks, as well as to tasks involving verbal comprehension (Sternberg 1983). His analysis of basic processes was similar to that of Spearman (1923), but somewhat more elaborate. He suggested (Sternberg 1983), at least in inductive reasoning (which is close, but not identical to fluid intelligence), components of encoding (Spearman’s apprehension of experience), inference (Spearman’s eduction of relations), mapping of higher order relations, application (Spearman’s eduction of correlates), comparison between stimuli, justification of an answer that was better than alternatives but imperfect, and preparation-response (estimated mathematically as a regression constant).

Sternberg, like Hunt, was critical of psychometric work for failing to elucidate the processes of intelligence. However, in retrospect, psychometric work lay the basis for much of the information-processing work that was to follow. For example, both Hunt and Sternberg used correlations of individual component scores (usually latencies) with psychometric test scores to validate whether particular identified processes were actually “intelligent” ones. Furthermore, it was one such correlation that eventually would lead Sternberg to seek alternative approaches to analysis. Thus, the various approaches seem best to be viewed as complementary, rather than one as “replacing” another, which was what the information-processing psychologists hoped for at the time.

Sternberg’s use of componential analysis culminated in his book on his triarchic theory of intelligence (Sternberg 1985a) and then dropped off as he pursued different approaches to intelligence. The componential approach had at least one advantage over the cognitive-correlates approach, in that the information-processing analysis was based on the actual item types that appeared in the current intelligence tests of the time (and that still appear today). So, at the least, intelligence researchers had claimed the relevance of the tasks to intelligence. However, the approach had many of the other negatives of the cognitive-correlates approach, plus, arguably, four additional ones.

First, because the items came from intelligence tests, the correlations of the information-processing component scores with intelligence-test scores could be viewed as less than surprising. Components of cognitive tasks can be expected to correlate with scores on the psychometric tests from which the tasks were drawn, even if the measurements are experimentally independent.

Second, the results of Sternberg’s (1977) research and his later research (see Sternberg 1983) highlighted the dubiousness of the claim that information-processing research was yielding single identifiable basic processes. Sternberg (1977) found that the information-processing score that had the highest correlation with tests of fluid intelligence was the regression constant, which Sternberg referred to as “preparation-response”. The component that was supposed to be the “throw-away” in the multiple-regression equation actually turned out to be the most important one, at least in terms of correlation with *g.* In all likelihood, the process involved something beyond preparation and response time. 

Third, in the Sternberg studies of analogical reasoning (Sternberg 1977; Sternberg and Rifkin 1979), the encoding component actually took longer for better reasoners. In other words, more intelligent participants spent more time in encoding so that they could operate more quickly on the encodings. In later research, Sternberg (1981) showed that more intelligent participants also spent relatively more time on global planning, or the planning one does before solving a problem, than on local planning, so as to make the actual solving of the problem more efficient. 

Both of these results—concerning encoding and global planning—made conceptual sense. Sternberg explained them in terms of metacomponential, or executive processing, part of which involved planning out one’s strategy for solving a given problem. However, the results called into question the practice of merely viewing faster reaction times as indicating greater intelligence. What appeared to matter most was not overall speed, but rather the allocation of resources so that component processes that needed more time obtained more time, and those that needed less time obtained less time. This made sense in terms of Sternberg’s theory, but not in terms of some of the other theories simply linking the speed of mental processing to intelligence.

Others beside Sternberg (1983) tried various kinds of componential analyses. Like Sternberg, Pellegrino and Glaser (1979) compared the cognitive-correlates and cognitive-components approaches and found the approach emphasizing components more congenial. For example, Mulholland et al. (1980) performed an elegant analysis of the solution of geometric analogies, finding underlying processes similar but not identical to those elucidated by Sternberg (1977). Pellegrino and Glaser (1980) showed how the analysis could be applied across different kinds of induction items. Snow and Lohman (1984, 1989) used similar analyses on a variety of inductive-reasoning items, and Snow et al. (1984) constructed a “topographical” (structural) model that attempted to integrate the various kinds of information processing. Carpenter et al. (1990) performed an elegant analysis of the Raven Progressive Matrices test (Raven 1938, 1986). They not only elucidated processes underlying the solution of the test but also showed how it was possible to construct a computer simulation that could do a credible job of solving the problems. Such analyses could be applied beyond reasoning problems. Pellegrino and Kail (1982) used similar techniques to analyze performance on spatial-relations tasks (see also Shepard and Metzler 1971).

Fourth, the processes that were supposed to be basic did not seem to be basic in any serious sense. For example, if preparation-response showed the highest correlation with *g,* it presumably was measuring more than just residual information processing at the beginning and end of problem solving. The encoding process, which increased in latency with age and ability, must have been quite complex to allow for identifying important elements of the problem, and inference also must have been a complex process: One needs to know on what information to focus and what information one can discard. None of the identified processes convincingly can be called “basic.

#### 3.2.3. The Working-Memory Approach

More recent work on working memory (Conway and Kovacs 2013, 2020; Ellingsen et al. 2020; Engle 2018; Engle and Kane 2004; Kovacs and Conway 2019a, 2019b), covered in other essays (see also Daneman and Carpenter 1983; Kyllonen and Christal 1990), suggests that working memory plays an important part in intelligence. This work has the advantage over much of previous cognitive-correlates work in that it is theoretically based. The correlations also have tended to be higher. However, the work suffers from most of the other problems—that, in the end, it relies on the notion that intelligence is what the tests test (Boring 1923). This approach also has the same problem as other approaches with respect to a key issue: What exactly is a basic process? Newell and Simon (1972) thought that they had identified elementary information processes. Since then, it has become, if anything, less clear what an elementary information process actually is.

Kovacs and Conway (2019a, 2019b) have proposed a very plausible theory of process interaction, by which *g* would reside in the interaction of executive processes, what Sternberg (1985a) calls “metacomponents”. Indeed, the Kovacs-Conway view is closely related to Sternberg’s view of general intelligence as residing in the metacomponents and their interactions (Sternberg 1985a; see also Sternberg 2021c; Sternberg et al. 2021b). In the Conway and Kovacs (2020) point of view, working memory and intelligence are related through fluid intelligence and through mechanisms of cognitive control in working memory. Ellingsen et al. (2020) view the relationship somewhat differently, with working memory functioning to retain information that is needed for cognitive processing and fluid intelligence working to discard information that is no longer needed. 

#### 3.2.4. Other Approaches

This list of approaches to elementary or basic processes is far from exhaustive. Another approach is that of complex problem solving (e.g., Greiff et al. 2013). Yet another is a cognitive-training approach (Chi 1978, 1992; Schneider et al. 2020; Sternberg et al. 1982). Further, much recent work has taken a neuropsychological approach (Haier 2020; Haier and Jung 2007; Jung and Haier 2007; Posner and Barbey 2020). There simply is not room to review all approaches here.

## 4. What Intelligence Is Varies in Part from Place to Place and Time to Time

All of the approaches described above face challenges. For example, Greenfield (2020) has shown that what properly might be called “intelligence” varies over both time and place (see also Berry 1974; Cole et al. 1971; Preiss and Sternberg 2005; Serpell 1974; Sternberg 2004). That is, a mental process is always and everywhere what it is—but the extent to which it can be classified as *intelligent* can differ across time and place. In other words, its operation is affected by the context in which it is performed.

On the one hand, the elements of *g* seem to be somewhat invariant. Almost a century after Spearman’s (1927) famous treatise on general intelligence, it still is unclear what *g* is. Theories continue to abound trying to figure it out (see essays in Sternberg 2020c). Sternberg (1985a) suggested it comprises a set of executive processes—what he called “metacomponents”—recognizing the existence of a problem, defining the problem, allocating resources to the solution of the problem, mentally representing the problem, formulating a strategy to solve the problem, monitoring problem solution, and evaluating solution of the problem after its solution is completed. Sternberg suggested that, regardless of the content of the problem, these processes would be needed, at some level, to solve it (see also Conway and Kovacs 2020; Kovacs and Conway 2019a). Similarly, working memory would seem to be involved in virtually any problem requiring intelligence. The relevance of the speed of mental processing is far less clear: Some problems require a quick solution, but others do not, and indeed, a quick solution may be detrimental to the solution of some problems.

### 4.1. The Inconstancy of the Processes of Intelligence over Time

The issue is that what constitutes intelligence, and what constitutes the many processes needed to solve problems requiring intelligence, are not constant. Rather, they change.

Consider, for example, mental computation. When the author’s father owned his own business, many years ago in the middle of the 20th century, he spent a considerable amount of time adding sums of numbers relevant to his inventory and payroll. He was very quick and accurate at such computations, which certainly enhanced his ability to succeed in his business. There were burdensome calculators available at the time but using them would have taken longer than did his mental arithmetic and would not have been much more accurate. It hence makes sense that, at that time, the *SRA Primary Mental Abilities* test, based on the work of Thurstone (1938) and Thurstone and Thurstone (1941), measured numerical ability by presenting test-takers with strings of addition problems with carrying. At the time, addition with carrying was an important skill for adaptive success in life. That, of course, was before easily portable hand calculators and then computers. Today, such skills may matter for some people, but scarcely would constitute anything close to a sufficient sampling of the full set of skills needed to be successful in solving mathematical problems in everyday life. Such problems no longer appear on contemporary intelligence tests.

Similarly, when the author of this article was in elementary school, he had a friend who was clever but a very poor speller. Today, maybe he would be referred to as someone living with dysgraphia. Spelling, unlike arithmetic, did not appear on the intelligence tests of the era. However, being a poor speller was perhaps even more baneful than being poor in arithmetic in its consequences for school performance. There were no computers, no spell checkers. Furthermore, whereas teachers might have made some allowances for children with dyslexia as having a specialized learning disability, few of them probably even had heard of dysgraphia. Many of the child’s teachers considered him just not to be very bright. Today, spelling skills are much less important for adaptation. We have computers and not only spell checkers, but also software that automatically corrects our spelling errors (sometimes, it overcorrects, putting in the wrong word when “thinking” it is correcting our spelling.) 

The number of skills that have become less important for adaptation is quite large. Hunting skills matter less to most of us, as do gathering and fishing skills. At one time, poor spatial–directional skills could lead to one’s demise if one accidentally entered enemy territory or left territory where one could find adequate food and shelter. (They still occasionally do, but much more rarely.) The author used to get lost all the time, sometimes painfully, in either large cities or expansive rural areas. Today, GPS usually will solve the problem of poor spatial–directional skills. Even London cab drivers today probably can get by with having mediocre directional skills. That is a real coup for those who want to make money but cannot find their way through the labyrinthine warrens of London streets.

So, how important are the mental processes of arithmetic computation, or spelling, spatial navigation, or hunting to intelligence? It depends on the context. However, one thing is clear. For many people, these skills are much less important than they would have been a generation ago. Furthermore, if one went back more generations, the difference might have been quite a bit greater, certainly for spatial-navigation, hunting, and gathering skills. At one time, if you could not hunt and gather, you might have perished. Your IQ-based skills, whatever they were, would not have helped you if you could not eat.

Whereas some skills have become less important over time, others have become more important. Consider two such skills: information retrieval and information evaluation.

When the author was young, there were a few major national newspapers in the United States—the *New York Times*, the *Herald Tribune*, and perhaps the *Wall Street Journal* for the business community. All of them carried pretty much the same news, presented with slightly different slants. The *Times*, for example, was slightly more left-wing, the *Tribune* and the *Journal*, more right-wing, but the difference was one of slant rather than of the news covered or of how it was presented. Similarly, two major U.S. national magazines, *Time* and *Newsweek*, covered pretty much the same news, the same way. *Time* was more conservative, *Newsweek* more liberal, but the difference was clearly limited to opinion in the editorial pages. The three major U.S. television networks for nightly news, CBS with Walter Cronkite, NBC News with Chet Huntley and David Brinkley, and ABC News with Howard K. Smith, similarly covered largely the same news, each with a slightly different slant. What was common to all these sources of news was the strict and societally responsible editing in which they engaged, or at least attempted to engage. 

That was then. Today, news better could be described as “news”. Each channel of news will present potentially different stories, different so-called “facts”, and of course, different points of view. However, many news outlets will not carefully distinguish, and some will not distinguish at all, between what is “news” and what is opinion. Their worldview will become the basis of what is covered in their news, and how it is covered. Those consumers of news who do not think analytically and reflectively will risk falling prey to lies and demagogues. 

Information-processing skills are far more important than they were in the past. In those days, one might go to a library for information, where a librarian might serve as a vetting source for books, magazines, and newspapers that already had been vetted. One might go to a carefully edited encyclopedia, such as the *Encyclopedia Britannica* or the *Encyclopedia Americana*, both of which had editors priding themselves on their meticulous screening of information. Or one might take one’s chance at a news stand, but the extreme sources of news, say, coming from the ultra-right or ultra-left wings, were clearly identifiable as such.

Those days are over. Moreover, many websites have specialists in increasing “click rates”, carefully vetting the news feeds sent to consumers to conform to their biases and prejudices, no matter how extreme. If one were not highly reflective about information retrieval, it would become possible, quite easily, to start thinking that everyone sensible thinks the same way one does oneself, except, of course, for people who are ignorant, unreasonable, or just plain stupid!

One needs far more skills in discerning and reflective information retrieval today because the vetting of information on the Internet ranges from careful and accurate, to nonexistent, to purposefully biased and misleading. One simply *must* learn how reflectively and objectively to evaluate the information obtained from the Internet or from other sources. Lack of reflective evaluation potentially leads one to indulge in and ultimately to become part of the echo chambers that have become so notorious in current society. In these echo chambers, groups of people of similar ideology come together and share stories, many of them patently ridiculous, of conspiracies that are not, and in all likelihood, could not be occurring.

Information retrieval and evaluation have become much more important. A third skill that has become much more important is information sharing—what information one should or should not share, and how one should share it, if indeed one chooses to do so. 

At one time, if we had some thoughts we wanted to share, we might share them among our close or not so close friends and perhaps with some people in our local neighborhood. We knew to whom we were talking and probably how to communicate in a reasonably effective way with them. Today, with the advent of social media, one sometimes does not know how far one’s messages will reach or whom they will reach. They may reach no one, or they may go “viral”. If the messages are not written carefully, or if they are messages that never should have been released in the first place, the messages can cause lasting damage both to ourselves and to others. How many people have lost jobs or opportunities for jobs or schooling because of postings carelessly shared on social media? The consequences of information sharing are, on average, far higher than they were some years back.

### 4.2. The Inconstancy of the Processes of Intelligence over Space

What is intelligent also can differ not only over time but also across space—from one culture to another. Thus, rank orders of individual differences in intelligence depend on where and when one lives. The adaptive requirements of life in different places are, simply, different.

There are many examples of how processes of intelligence, or at least their instantiations, change as a function of culture and also of the temporal period of the particular culture. These examples have in common that the adaptive requirements for success and even survival differ from one place to another.

In a study of the practical side of intelligence of children in rural Kenya (Sternberg et al. 2001), it was found that children who performed well on a test of practical knowledge of natural herbal medicines that could be used to combat parasitic illnesses tended to score lower on tests of conventional intelligence than did children who performed more poorly on the tests of knowledge of herbal medicines. In other words, there were negative correlations between the two types of tests. 

It might seem that a test of how to recognize parasitic illnesses and treat them would have little to do with intelligence, as we know it. However, it is worth thinking carefully about this. Intelligence is the ability to adapt to the environment, a definition recognized by many experts on intelligence going way back in time (Binet and Simon 1916; Colvin et al. 1921; Wechsler 1940). For these children, a test of procedural knowledge about dealing with parasitic illnesses is about as central to adaptation to the environment as a test could be. The parasitic illnesses they face, such as malaria, trichuriasis (whipworm infection), schistosomiasis, hook worm infection (which can cause severe anemia), and others, are extremely debilitating and can be fatal. 

Children suffering these diseases often are stunted in their cognitive development (Sternberg et al. 1997) because they can experience brain fog, are distracted from scholastic achievement by their disease, and often have poor attendance in school as a result of the illness. Moreover, the illnesses tend to be recurrent. Children place dirt into their mouths, resulting in repeated Trichuris worm infection, or walk into contaminated water, resulting in schistosomiasis infection. Because modern Western medications often are not available, knowledge of the natural herbal medicines is important knowledge to have. Furthermore, the medications actually work.

It might seem to some readers that this kind of knowledge, even if it is adaptive, is quaint, only applying in less developed, non-industrialized cultures. However, by far the biggest killer disease of all times has been malaria, which has killed an estimated 50 billion people over time (Business Tech 2014). Malaria still kills people in much of the world.

At the time the work in rural Kenya first was reviewed, a reviewer of the work was skeptical that the kinds of skills studied had anything to do with intelligence as adaptation outside such developing nations. However, the COVID-19 pandemic has shown how relevant such knowledge is, albeit for a different presenting disease. As of the date this article is being finalized (12 May 2022), COVID-19 has killed over 6 million people. That is a far cry from the 50 billion that malaria may have killed, but by any standard, it represents a cruel pandemic. Furthermore, although no disease is entirely preventable, there are steps people can take, while waiting to be vaccinated or even after vaccination, to reduce or even minimize risk to themselves and others, such as wearing securely fitted masks, social distancing, washing hands, avoiding crowds, and the like. 

Curiously, some of the skills children in rural Kenya need to preserve their and others’ lives are now important world over. The content is different, but the underlying skills of learning and practice are probably not so different. Just two years ago, almost no one would have given these skills even a second thought as critically important to adaptation to the environment in the developed world. 

Other skills matter in some cultures but not in others. For example, among Yup’ik Native Americans in small Alaskan fishing villages, skills such as hunting and ice-fishing are essential to adaptation to the environment, and many related skills once were important where many of us live, generations back (Grigorenko et al. 2004). Many of the skills of these Native American also seem irrelevant. However, when we lose electric power in our homes, skills we usually do not need come to the fore; when we become trapped in a snowstorm while driving in a remote region with no immediate prospect of outside help, survival skills can mean the difference between life and death.

The number of cultural differences in adaptive requirements is large (Ang and Van Dyne 2015; Cohen and Kitayama 2020). The mental processes needed to deal with these challenges probably are overlapping, but they also probably are non-identical. Perhaps more importantly, even to the extent that there is overlap in processes, the situations in which the processes need to be deployed are quite different (Sternberg 2004). Furthermore, there is evidence that the same deep-structural challenges presented in different contexts often elicit different solution strategies (Bronfenbrenner and Ceci 1994; Ceci and Bronfenbrenner 1985; Ceci and Roazzi 1994; Nuñes 1994). 

## 5. Breadth of Information Processing Underlying Intelligence

Many, perhaps most researchers in the field of intelligence, are comfortable with the set of processes constituting the model of intelligence proposed by Carroll (1993), perhaps as modified by McGrew (2005) and others who have developed the so-called CHC (Cattell–Horn–Carroll) expansion of Carroll’s model. Using the CHC model, some researchers have sought to expand upon this set of processes. Seven broad factors of the CHC model include some mental processes, such as auditory processing, crystallized intelligence, fluid reasoning, long-term retrieval, processing speed, short-term memory, and visual-spatial thinking.

One of the earliest expansionists was Thorndike in his work on social intelligence (Thorndike and Stein 1937). The initiation of the concept of emotional intelligence also expanded the range of thinking about intelligence (Mayer et al. 2004; Rivers et al. 2020). Others have explored multiple intelligences (Gardner 2011; Kornhaber 2020), successful intelligence (Sternberg 2020e), collective intelligence (Malone and Woolley 2020), leadership intelligence (Boyatzis 2020), cultural intelligence (Ang et al. 2020), practical intelligence (Hedlund 2020), social intelligence (following Thorndike; Kihlstrom and Cantor 2020), consumer and marketer intelligence (Sujan and Sujan 2020), and mating intelligence (Geher et al. 2020). Most of these kinds of intelligence have not been subject to satisfactory empirical validation, although Gardner’s theory has been with less than supportive results (Visser et al. 2006). An older test of Sternberg’s to measure intelligence according to his earlier triarchic theory also had issues (Brody 2003), but later tests appear to have been considerably more successful (Sternberg 2010; Sternberg and Rainbow Project Collaborators 2006; Sternberg and Sternberg 2017; Sternberg et al. 2017, 2019, 2020, 2021a). For example, tests of creative and practical skills, as well as of scientific-reasoning skills, were shown to be, at best, weakly correlated with tests of general intelligence, and in some of these cases, negative correlated (see also Sternberg et al. 2001).

Only two of these accounts, the theories of multiple intelligences and of successful intelligence, purport to be broad theories of intelligence, per se. How one conceptualizes these various accounts affects how one views the processes of intelligence. There are at least four possibilities.

1. **Broader theories of kinds of intelligence are not really theories of intelligence at all.** On this view, the theories use the word “intelligence” but are really theories of something else, perhaps of “skills”, “abilities”, or “aptitudes”, etc. In this case, the question of whether the processes of intelligence need to be broadened simply does not apply. One recent review of intelligence (Deary 2020) does not appear to view these various theories of intelligence as serious contenders for a place in the intelligence literature. Hunt (2011) took a similar view.
2. **Broader theories perhaps are theories of kinds of intelligence, but these kinds of intelligence are, at best, peripheral in the study of intelligence.** In his textbook on intelligence, Mackintosh (2011) viewed the broader theories of intelligence as interesting but as somewhat peripheral.
3. **Broader theories need to be taken seriously as central to the field of intelligence, just as does general intelligence.** This viewpoint probably would fairly represent that of Sternberg (2020c, 2020d) and of some of the authors in these volumes.
4. **Broader theories need to subsume and possibly replace existing theories of intelligence, which simply are too narrow.** This view would be that of Gardner (2011) and of Sternberg (2021b). Gardner and Sternberg would argue that traditional theories have been too narrow and have “missed the boat” in terms of understanding the abilities that intelligence should encompass. Gardner’s theory, however, has lacked traditional empirical support.

For some debates, this would be the place to present the evidence in favor of and opposed to various points of view. That cannot be performed here, because the issue is definitional and to a large extent meta-theoretical. It is not subject to empirical resolution. One could design a study and then could test a theory within any of these meta-theoretical frameworks, (e.g., Visser et al. 2006), and then argue that that particular theory is or is not valid. However, in this case, even if the work shed doubt on a theory such as that of multiple intelligences, it would not resolve the question of whether a broader framework is needed. For example, it would not be a great stretch of the imagination to present a theory of multiple intelligences that allowed for intercorrelations among the multiple intelligences. Indeed, to the extent that any test of any intelligence is not likely to be pure, and to the extent that the multiple intelligences probably have to work together, one could argue that the correlations among tests represent not a failure of the theory but rather represent the interactions of subsets of the intelligences in any single test, interactions that Gardner (2011) himself would predict.

In the end, there probably will continue to be different views on how far the notion of intelligence as adaptation to the environment should be extended. The differences have become somewhat ideological, as have what once were reasoned differences within the political spectrum of the United States and other countries.

Perhaps the narrow conception of intelligence may be reaching its expiration date. However, it is not clear that any conception of intelligence will last forever. The advantage of some of the broader views is that they encompass the narrower views (Sternberg 2020e). Even Gardner (2011) would say that *g* would be comprised of elements in his theory of multiple intelligences. The challenges the world faces are no longer those of Spearman’s (1927) or Binet and Simon’s (1916) world. The world today, or at least, humanity and millions of species beyond it, face existential threats that did not exist before (Sternberg 2021b). However, it is unclear where both theorizing and the world in which it occurs will go. 

## 6. Conclusions

The information-processing paradigm has been a partially successful attempt to better understand the nature of intelligence. In many respects, as Conway and Kovacs (2020) suggest, the work fulfills the hopes of Cronbach (1957) for an integration of differential and experimental psychology. However, early hopes for its being, somehow, a definitive answer to the problems of the psychometric paradigm (e.g., Sternberg 1977) were unrealistic and have not been borne out. Moreover, recent work, especially on working memory, have shown that the information-processing approach still has a lot of life in it. Exactly where the approach should go is a matter of dispute (e.g., Deary and Sternberg 2021). Should the study focus on simple and choice reaction time, inspection time, inductive reasoning, working memory, or perhaps some other type of information processing (as reviewed in Sternberg 1985b and more recently in Ellingsen et al. 2020)? There probably is no one answer. What is clear is that the study of information processing in intelligence has revealed how a paradigm can be very enlightening, and yet, in the end, pose as many new problems as the number of old ones it addresses, and perhaps even resolves. So, the information-processing approach filled in a large gap left by psychometric theories, only to create new gaps that have been addressed by neuropsychological, cultural, and other approaches (Sternberg 2020c). 

Table 1 summarizes some of the problems researchers have encountered with the search for basic processes of intelligence. The problems are numerous and diverse. They suggest that while process analysis may be productive, the search for basic or elementary processes (Newell and Simon 1972) may not be.

Many psychologists long have hoped that there was going to be a single approach that would provide *the* answer to the nature of intelligence, and in particular, the basic processes that underlie it. Certainly, that was the hope of Sternberg (1977), drawing on Spearman (1923). Sternberg proposed that componential analysis somehow would yield *the* basic processes underlying intelligence. That hope was too ambitious. Rather, information-processing approaches have complemented other approaches in yielding valuable but nondefinitive answers. There has been, at least so far, no one paradigm that has provided final answers. Even biological approaches have not been definitive. These approaches have been championed by Richard Haier (2016), who has suggested that intelligence is at its root, biological. Haier has proposed a biological theory and there are other biological theories as well (see, e.g, Barbey et al. 2021). None of these theories would account adequately for a phenomenon as simple as why people so often act against their own interests, such as when they contribute to global climate change. The answer is not in finding a single encompassing explanatory unit, such as the factor, or the elementary or basic process, but rather in figuring out how the converging operations of multiple approaches (Garner et al. 1956) can elucidate the incredibly complex phenomenon of intelligence. Figuring out the nature of intelligence requires intelligence, but it also requires epistemic humility, as elucidated by Plato in *The Apology* (20e–23c)—the realization that we and our insights are often not as all-encompassing as we might hope they would be.

## Figures and Tables

**Table 1:** Why and how the search for basic processes of intelligence has not converged.

Problem	Example
We do not know how to find truly basic processes.	Investigators have used psychometric, biological, information-processing, and other analyses to uncover basic processes of intelligence, but the results have not converged convincingly either within or between methods.
If researchers discovered the basic processes of intelligence, they would not have the empirical operations to identify them truly as basic.	Many processes have been identified, but there has been no compelling demonstration of a method to label them as “basic”.
Many analyses depend on correlational methods, but correlations do not necessarily imply causation.	The fact that reaction times, for example, correlate with psychometric *g* does not in any way conclusively show that the processes are basic.
Researchers often do not know that a given process labeled as corresponding to a reaction time or error rate is actually the, or even a, correspondent process. The label may be wishful thinking.	Jensen (1998) claimed to measure the speed of neuronal conduction through his choice of reaction-time measure, but never showed that his experimental operation actually corresponded to a measure of speed of neuronal conduction.
Correlations of information-processing measures with scores on psychometric tests tend to be modest, or moderate at best.	Even if the correlations were psychologically meaningful, at their obtained magnitudes, it would not mean that they were indicative of basic processes.
The information-processing measures were originally designed to provide a causal understanding of intelligence, but their “validity” then often has been determined on the basis of correlations with the indices they were supposed to explain.	Sternberg (1983) set out to “explain” scores on fluid-ability tests, but he then interpreted as meaningful those component scores that showed significant correlations with those same psychometric tests he sought to explain. Moreover, the highest correlation was for the preparation-response component (the regression constant).
The correlations also are less than meaningful because there is no “true” correlation. The correlation will depend on the population, the task, and the situation in which the task is administered, as well as their interactions (Sternberg 2021c).	Meta-analyses have revealed a wide range of correlations between information-processing tasks and psychometric tests, depending on the population and circumstances of administration.
Meta-analyses solved none of these problems, because they too, for the most part, have been correlational. An analysis of many correlations is still correlational.	Meta-analyses of inspection time have helped to organize correlations, but they have never found any “true” single correlation, because there is none. At best, one can find a wide range.
There are too many “winners”. Large numbers of information-processing tasks correlate significantly and meaningfully with psychometric tests.	Most cognitive tasks show some correlation with psychometrically measured intelligence, as follows from Spearman’s (1927) theory. Some correlations, however, are extremely modest or negative (Sternberg et al. 2001).
Broader theories of intelligence, although in various states of validation, suggest that even if correlations are found with general intelligence, those correlations may apply only to a limited range of what meaningfully could be called “intelligence”.	Tests of emotional intelligence show correlations with a wide range of information-processing tasks and real-world behaviors (Rivers et al. 2020), and we have no basis for excluding this kind of intelligence (among others) from any analysis of “basic” processes.

## Data Availability

Not applicable.
